# A cross-sectional study exploring attitudes of first year medical students towards psychiatry and factors they identified that would help stimulate their interest

**DOI:** 10.1192/bjo.2021.412

**Published:** 2021-06-18

**Authors:** Isabel Mark, Victoria Fernandez Garcia De Las Heras

**Affiliations:** South West London and St George's Mental Health Trust, St George's University of London

## Abstract

**Aims:**

Psychiatry has had long standing recruitment difficulties. Many efforts have been made to explore strategies that encourage interest in the specialty, with early university experience being an important factor in ultimate career choice. The Royal College of Psychiatrists ‘Choose Psychiatry’ guidance for medical schools outlines four key areas of focus: teaching excellence, placement quality, leadership and enrichment activities, with other research reporting similar conclusions. The aim of this study was to assess attitudes towards psychiatry amongst first year medical students, examine what input they would welcome from psychiatrists at this stage of their career and consider if their wishes are in keeping with the ‘Choose Psychiatry’ guidance.

**Method:**

All first-year medical students at St George's University of London were approached in October 2019 and offered the opportunity for early psychiatry exposure. 60 students were recruited. Data were collected in November 2019 via an online questionnaire comprising of baseline demographics, the 30-item Attitudes Towards Psychiatry questionnaire (ATP-30) and a free-text question asking what students would like from psychiatry at this stage of their education. Quantitative data were analysed using Excel, whilst qualitative data were analysed thematically.

**Result:**

The mean ATP-30 score was 113.83 (SD 12.57, range 70-135). Gender, ethnicity and religious background were not associated with a change in ATP score. Undergraduates’ attitudes were more positive than those of postgraduates (independent t-test revealed a p-value of 0.087). Seven themes were identified outlining what students wanted from psychiatry, the most prominent being: (a) learning about the lifestyle of a psychiatrist and finding a role model, (b) exploring the patient perspective, (c) exploring the interaction between psychiatry and specialities and (d) having an opportunity to develop communication skills.

**Conclusion:**

The findings demonstrate higher ATP-30 results than previous literature has reported, potentially due to mental health awareness campaigns in recent years. As undergraduates were found to have a higher mean score, targeting them for additional psychiatry contact may be beneficial. Themes identified by students in this study support ‘Choose Psychiatry’ guidance, whilst also highlighting the potential for combining some psychiatry with other specialties in the curriculum. Integration with communication skills teaching might help engage those not initially psychiatry-inclined. Further study will be required to establish whether implementing the suggested strategies can lead to improvement in student ATP-30 scores and ultimately increased recruitment rates.

